# CD4 CTL, a Cytotoxic Subset of CD4^+^ T Cells, Their Differentiation and Function

**DOI:** 10.3389/fimmu.2017.00194

**Published:** 2017-02-23

**Authors:** Arata Takeuchi, Takashi Saito

**Affiliations:** ^1^Laboratory for Cell Signaling, Department of Immunology, RIKEN Center for Integrative Medical Sciences, Yokohama, Japan; ^2^Graduate School of Medical and Dental Sciences, Niigata University, Niigata, Japan; ^3^WPI Immunology Frontier Center, Osaka University, Suita, Japan

**Keywords:** CD4^+^ T cell subset, CD4 CTL, differentiation, antiviral immunity, inflammation, eomes, class I-restricted T cell-associated molecule, T cell activation

## Abstract

CD4^+^ T cells with cytotoxic activity (CD4 CTL) have been observed in various immune responses. These cells are characterized by their ability to secrete granzyme B and perforin and to kill the target cells in an MHC class II-restricted fashion. Although CD4 CTLs were once thought to be an *in vitro* artifact associated with long-term culturing, they have since been identified *in vivo* and shown to play important roles in antiviral and antitumor immunity, as well as in inflammation. Functional characterization of CD4 CTL suggests their potential significance for therapeutic purposes. However, in order to develop effective CD4 CTL therapy it is necessary to understand the differentiation and generation of these cells. Although the mechanisms regulating development of various CD4^+^ Th subsets have been clarified in terms of the cytokine and transcription factor requirement, the CD4 CTL differentiation mechanism remains elusive. These cells are thought to be most closely related to Th1 cells secreting IFNγ and regulated by eomesodermin and/or T-bet transcription factors for their differentiation. However, our studies and those of others have identified CD4 CTLs within other CD4^+^ T cell subsets, including naïve T cells. We have identified class I-restricted T cell-associated molecule as a marker of CD4 CTL and, by using this marker, we detected a subset of naïve T cells that have the potential to differentiate into CD4 CTL. CD4 CTL develops at sites of infections as well as inflammation. In this review, we summarize recent findings about the generation of CD4 CTL and propose a model with several differentiation pathways.

## Introduction

Naive CD4^+^ T cells differentiate into various effector T cell subsets characterized by their capacity to produce specific cytokines in order to promote various types of immune responses. Th1 and Th2 cells, which can produce IFNγ and IL-4, respectively, were the first described (1). Subsequently, various Th subsets with different functions have been reported, such as pro-inflammatory Th17 cells, follicular helper T cells (Tfh), and regulatory T cells (Treg), and their individual features have been clarified. Functional differentiation into the different Th subsets is induced upon T cell receptor (TCR) stimulation by peptide–MHC and is regulated by environmental factors. Particular combinations of cytokines induce expression of master transcription factors such as T-bet, GATA-3, RORγt, Bcl6, or Foxp3, which induces CD4^+^ T cells to differentiate into Th1, Th2, Th17, Tfh, or Treg cells, respectively (2). These Th subsets “help” to create optimal conditions for other lymphocytes to enable their function during different types of immune response. More specially, the cytokines produced not only can promote phagocytic activity, generation of CD8 cytotoxic T cells (CTL), antibody production, and pro-inflammatory responses but also can function to suppress the response. CD4 CTLs were identified as an unexpected CD4 subset with cytotoxic function. These cells can secrete cytotoxic granules containing granzyme B and perforin and directly kill target cells in an antigen (Ag)-specific fashion upon direct contact. Similar to the other CD4 T cell subsets, it should be possible to identify CD4 CTLs by some specific marker proteins or transcription factors (3). We will discuss here what is known about CD4 CTL, with particular focus on the differentiation and function of this subset of T cells.

## Identification of CD4 CTL

CD4^+^ T cells recognize Ag peptide in the context of the MHC class II (MHC-II), and CD4 CTLs are no exception. CD4 CTL target cells are restricted to class II-expressing antigen-presenting cells (APC) such as dendritic cells, macrophages, or B cells. MHC-II-restricted cytotoxic T cells were first identified decades ago in alloreactive responses (4, 5). Since then, many reports have described T cell lines and clones corresponding to CD4 CTL from both human (6, 7) and mouse (8, 9). Although these cells showed MHC-II-restricted Ag-specific cytotoxic activity, the possibility that this activity was an *in vitro* artifact resulting from long-term *in vitro* culture could not be excluded. Recently, CD4 CTLs have also been identified among PBLs of humans, especially under conditions of chronic viral infections, such as human cytomegalovirus (10, 11), human immunodeficiency virus 1 (11, 12), and hepatitis virus (13). CD4 CTLs have also been found in mice infected with gamma-herpes virus (14). These reports suggest that the T cell lines and clones derived from long-term culture might correspond to the *in vivo* situation in which CD4^+^ T cells are exposed to Ags for a long time upon chronic virus infection. In fact, during influenza virus infection, influenza-specific cytotoxic activity of CD8 CTLs is impaired in the chronic phase of infection, and CD4 CTLs can function instead (15). However, Swain et al. showed that CD4 CTLs are also observed in an acute phase influenza virus infection model (16). Although it is still unclear whether the CD4 CTLs generated in chronic and acute influenza infection have the same characteristics, these results indicate that CD4 CTL can be generated during both chronic and acute virus infections. CD4 CTLs have been detected mostly in virus infection models, suggesting that one of the main functions of CD4 CTLs is antiviral immunity. CD4 CTLs have also been detected during antitumor responses (17, 18) and chronic inflammatory responses such as autoimmune diseases (19, 20). In these cases, CD4^+^ T cells are also continuously exposed to Ag. These reports clearly indicate that CD4 CTLs are generated under various inflammatory conditions, and that these cells can exhibit functions complementary to CD8 CTLs *in vivo*.

## CD4 CTL Target Cells

CD4^+^ T cells recognize Ag peptides presented by MHC-II that are phagocytosed and processed in the endosomes of APC. These peptides are typically derived from outside the cell as exogenous Ags, but endogenous self-peptides can also be presented through the autophagy process (21, 22). B cells present Epstein-Barr virus (EBV) Ag peptide by this pathway (21), and CD4 CTLs directly target the viral peptide–MHC complex and kill the B cells (23, 24). Thus, CD4 CTL can function in immune surveillance of APC. Furthermore, not only APC but also cells that do not normally express MHC-II can become targets for CD4 CTLs. For example, IFNγ treatment or irradiation induces the expression of MHC-II on the surface of epithelial or tumor cells (17, 18, 25, 26), and lung epithelial cells express MHC-II after infection with parainfluenza or *Mycobacterium tuberculosis* (27, 28). CD4 CTLs may recognize viral Ags presented by MHC-II on these epithelial cells and lyse them as target cells. It is well known that many viruses such as EBV, CMV, and HSV try to escape from CD8-mediated cellular immunity by downregulating the expression of MHC-I on the surface of infected cells through inhibition of the TAP transporter and/or proteasome degradation pathways (29, 30). In order to overcome this virus escape mechanism and prevent viral expansion, infected target cells may present viral Ags on the induced MHC-II. As a result, CD4 CTLs can lyse the target cells in a class I-independent, class II-dependent manner. On the other hand, we have to consider that the evidence for such class II-restricted killing has come mainly from experiments using peptide-pulsed transformed B cells or splenocytes as target cells. It is still debated how frequently class II-induced non-APC are killed by CD4 CTLs *in vivo*.

## Markers for CD4 CTL

Markers specific for CD4 CTL have not been identified yet, but some markers related to cytotoxic functions of CD8 CTL or NK cells can be used. CD8 CTLs release cytotoxic granules directed toward the target cells, which are secreted from secretory lysosomes that are translocated to and fused to the plasma membrane (31). After secretion of granules, proteins localized in lysosomes such as lysosome-associated membrane glycoproteins (LAMPs), LAMP-1 (CD107a) and LAMP-2 (CD107b) are abundantly expressed on the cell surface. These molecules are markers for degranulation, and CD4 CTL can be identified by natural killer group 2 (NKG2A), which is a member of C-type lectin receptor family and forms a heterodimer with CD94. Its ligand is the non-classical MHC class Ib molecule HLA-E (or Qa-1^b^ in mouse) that presents a nonapeptide derived from MHC-Iα chain (32, 33). In addition to NK and CD8^+^ T cells, CD4 CTLs also express NKG2A in effector sites (34, 35). Interestingly, NKG2A may transduce an inhibitory signal, which may contribute to the dysfunction of Ag-specific CTLs during chronic viral infections and in tumors (36). NKG2D, another family member, is also a marker for CD4 CTL (35, 37). This receptor forms a homodimer on the surface of NK cells and CD8^+^ T cells and induces activation signals for cell polarization and degranulation (38). Thus, these molecules as CD4 CTL markers are commonly shared with NK cells and CD8 CTLs, indicating that CD4 CTLs have similar characteristics/functions.

We recently reported that the class I-restricted T cell-associated molecule (CRTAM) is another marker of CD4 CTL (39). We identified CRTAM originally as an early activation marker of NK and CD8^+^ T cells. CRTAM binds to its ligand, cell adhesion molecule-1 (40, 41). The heterotypic interaction is important for the maturation of CD8^+^ T cells during immune responses in lymph nodes (42). We also found that a small fraction of activated CD4^+^ T cells express CRTAM and that only CRTAM^+^ CD4^+^ T cells develop into effector CD4^+^ T cells with cytotoxic function (39). Cytotoxic activity is acquired after incubation of CRTAM^+^ CD4^+^ T cells with IL-2. These results indicate that lineage development of CD4 CTL is dictated to some extent during an early stage after T cell activation and that CRTAM is an early marker of CD4 CTL. Because CRTAM is only transiently expressed upon TCR stimulation, its usage as a CD4 CTL maker to detect and trace the cells *in vivo* is limited.

Downregulation of costimulatory receptors such as CD27 and CD28 may also be markers on CD4 CTLs (12). In general, cells losing the expression of CD27/28 have been characterized as Ag-experienced, further differentiated cells. Conversely, the expression of CD57 (HNK-1/Leu-7) is upregulated in cells with cytotoxic activity (43, 44), particularly in both human (10, 45) and mouse (14) chronic infection models. In a mouse acute infection model of influenza virus, CD4 CTLs are detected in both the CD27^+^ and CD27^−^ populations (46), and the majority of Eomes^+^ CD4 CTL expresses CD27 in an experimental autoimmune encephalomyelitis (EAE) model (47), indicating that these molecules do not necessarily represent authentic markers for CD4 CTLs. These data suggest that CD4 CTLs are enriched in further differentiated T cells.

## Differentiation of CD4 CTL

A number of studies on the differentiation of CD4 T cells into CD4 CTLs have revealed various cellular origins. CD4 CTL can apparently develop from Th0 (48, 49), Th1, Th2 (50), Th17 (46), and Treg (51) effector subsets. However, CD4 CTL derived from Th1 (or Th1-like) cells represent the majority of CD4 CTLs, which produce IFNγ alone or together with other cytokines such as TNFα and IL-2 (10, 12, 23, 52). It is well known that the transcription factor T-bet functions as the master regulator of Th1 differentiation and induces IFNγ production. T-bet also induces the expression of granzyme B and perforin, which are required for CD8 CTL activity (53). In an acute influenza virus infection model, the expression of T-bet was predominantly observed at the effector sites, suggesting that T-bet also promotes CD4 CTL differentiation (16). Interestingly, it has also been shown that the expression of eomesodermin (Eomes) but not T-bet is increased in the secondary effector phase and may play a critical role in the late stage of viral infection (54). CD4 CTLs may be regulated by both of T-bet and Eomes, depending on their maturation stage.

It is noteworthy that CD4 CTLs have been mostly observed in virus infection models. Since virus infection typically leads to a Th1-skewed condition, CD4^+^ T cells may differentiate into Th1 cells upon exposure of IL-12 or IFNγ produced by APC. CD4 CTLs are clearly induced during the antiviral responses to help clear the virus. However, it was also reported that IFNγ is not required for the induction of cytotoxic activity because IFNγ-deficient CD4^+^ T cells exhibit Ag-specific cytotoxic activity (16). Thus, whether Th1-skewed conditions are essential for induction of CD4 CTL still remains to be clarified.

Other reports showed that the cytotoxic activity of CD4 CTLs is enhanced when T cells are incubated under non-skewed (Th0) conditions rather than under Th1-skewed conditions (49). IL-2 signaling is essential for induction of CD4 CTL, and low doses of Ag could induce cytotoxicity more efficiently. IL-2 induces Eomes expression, which in turn promotes the expression of IFNγ and cytotoxic granules (55, 56). Interestingly, Eomes rather than T-bet contributes to CD4 CTL function in these cases (47, 57, 58). Furthermore, it is noteworthy that TCR signal strength affects the differentiation of effector cells and T cell polarization (59), and it is possible that CD4 CTL differentiation is similarly regulated.

CD4 CTLs can also be identified among intraepithelial lymphocytes (IEL) in the gut (60–63). Retinoic acid and TGFβ signaling terminates the expression of ThPOK and conversely upregulates the expression of both RUNX3 and T-bet. It was shown that these dramatic changes in transcription factor expression are required for reprograming of CD4^+^ T cells and their maturation to functional CD4 CTL. Furthermore, these cells seem to be derived from intestinal Treg cells in lamina propria (51). It is still unclear how much this reprogramming mechanism is involved in CD4 CTL differentiation during virus infection.

Recently, the relationship between CD4 CTL and Tfh cells has been shown. CD4 CTLs express high levels of Blimp1 and low levels of Bcl6 (64). The balance of these transcription factors is critical for inducing Tfh cells. However, in contrast to Tfh cells, CD4 CTL induction is suppressed by Bcl6 and TCF1, suggesting that the development of CD4 CTL and Tfh is reciprocally regulated.

As described above, we identified CRTAM as an early CD4 CTL marker. Initially, it has been reported that CRTAM^+^ CD4^+^ T cells are high producers of IFNγ and IL-22, and that CRTAM regulates T cell polarity and effector function (65). We found that CRTAM^+^ activated CD4^+^ T cells express high level of Eomes and generate CD4 CTL efficiently after cultivation with IL-2 under non-skewed conditions (39). We speculate that populations of naïve CD4^+^ T cells contain some CRTAM^+^ T cells at early stage of activation that have the potential to differentiate into CD4 CTL. Interestingly, CRTAM^+^ T cells can differentiate into Th1- or Th2-like cells under the respective skewing conditions, but importantly they still retain cytotoxic activity. These results indicate that CRTAM expression can induce cytotoxic activity regardless of cytokine environments. Accordingly, a study using CRTAM-transgenic (Tg) mice showed that CRTAM-mediated intracellular signaling induces Eomes expression and efficient differentiation to CD4 CTL.

By analysis of the CRTAM promoter it has been shown that AP-1 and ZEB1 can positively and negatively regulate CRTAM expression, respectively (66, 67), but the precise mechanism of CRTAM induction during *in vivo* immune responses remains unclear. Since different modes of T cell stimulation such as by Ag-APC or anti-TCR mAbs induce different levels of CRTAM, signal strength and environmental conditions at the time of stimulation, such as the cytokine environments, may affect CRTAM expression. CRTAM protein is degraded by the proteasome in the absence of stimulation. Even in CRTAM-Tg mice, the T cells express CRTAM on the cell surface only transiently and only upon stimulation (39). Thus, transient expression of CRTAM is tightly regulated both transcriptionally and posttranslationally. This suggests that even transient signal transduction from the cell surface CRTAM may be sufficient to induce differentiation to CD4 CTL.

**Figure 1:**
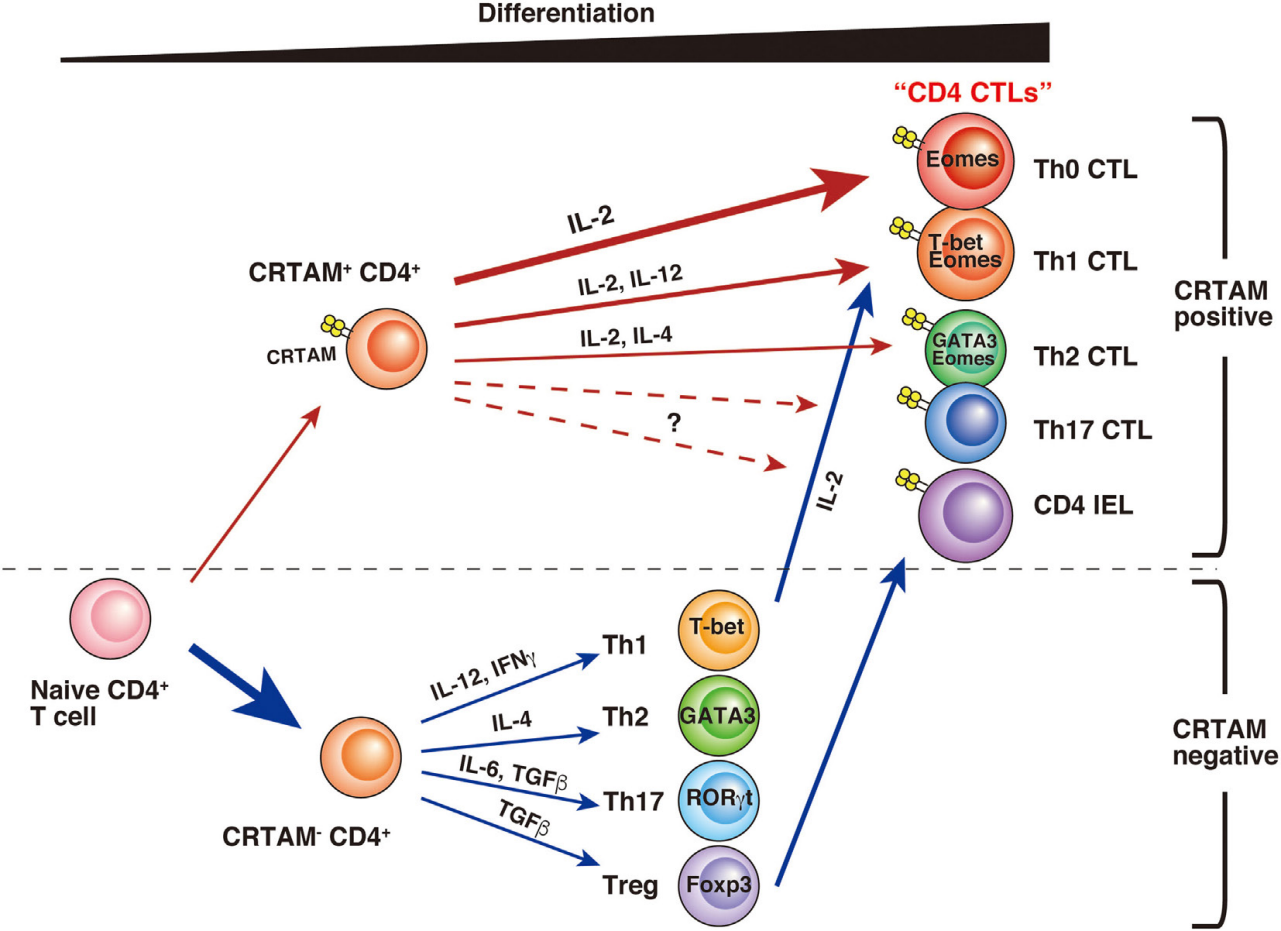
**A model of CD4 CTL differentiation**. After T cell receptor stimulation, a small fraction of naïve CD4^+^ T cells express class I-restricted T cell-associated molecule (CRTAM). CRTAM^+^ CD4^+^ T cells have the potential to differentiate into CD4 CTLs, which gain cytotoxic activity after incubation with IL-2 (Th0 CTL). Under the cultivation in the skewed conditions for each Th subset, they differentiate into Th1- or Th2-like cells with cytotoxic function (Th1 CTL, Th2 CTL). On the other hand, the majority of CRTAM^−^ CD4^+^ T cells can differentiate into various Th subsets based on environmental cytokines. Th1 polarized CD4^+^ T cells are known to show cytotoxic activity, and it has recently shown that intestinal regulatory T cells (Treg) can convert to cytotoxic CD4 intraepithelial lymphocytes.

CD4 CTLs seem to be derived from various types of CD4^+^ T cells, and several differences have been observed during their differentiation (Figure 1). Whereas a part of the cell population already has CD4 CTL potential from the early stage of activation, others may gain activity only after further differentiation. Although precise analysis is necessary to determinate whether these CD4 CTLs are the same subset, it seems likely that CD4 CTLs have several pathways of differentiation, each with different precursor cells. “CD4 CTL” should be general term used to describe “CD4^+^ T cells with cytotoxic activity” rather than one defining a uniform subset. Furthermore, several questions still remain about the relationship between CD4 CTL functions and CRTAM expression. First, it is still unclear whether all CRTAM^+^ CD4^+^ T cells can gain cytotoxic activity. Second, although CRTAM expression is downregulated within 48 h after stimulation, the potential to express is preserved, because all (or the majority) of cells express CRTAM again upon reactivation (39, 65). These results are slightly controversial and require further analysis to precisely determine the extent of plasticity of these cells. Third, in acute influenza virus infection, CD4 CTLs show Ag-specific cytotoxic activity that correlates with CRTAM expression. However, it is still an open question whether memory CD4 CTLs derived from naïve CRTAM^+^ CD4^+^ T cells are observed in sites of inflammation and have Ag-specificity. Clarification of these issues will promote the understanding of the function and differentiation of CD4 CTLs.

## *In Vivo* Function of CD4 CTL

CD4 CTLs with cytotoxic activity are mainly localized in peripheral tissues. In mouse models, influenza virus infections are restricted to the lung, and CD4 CTLs are also observed in this area. Under these conditions, not only APC but also infected epithelial cells can present Ag peptide on MHC-II, and CD4 CTL can recognize these cells and contribute to virus clearance. CRTAM expression can efficiently enhance CD4 CTL differentiation and probably also contributes to retention of CD4 CTL in the lung because lung epithelial cells highly express CRTAM ligand (68). Cytotoxicity by CD4 CTL becomes more effective when CD8 CTL activity is impaired during infections in association with virus escape strategies.

CD4 CTLs also have the potential to exacerbate autoimmune diseases. They contribute to the induction of intestinal colitis, and CRTAM is involved in the differentiation and residency of these T cells in the gut (39, 69). Indeed, CD4 CTLs are critically involved in the induction of colitis since colitis induction is reduced in granzyme B-deficient mice. Although it is still unclear whether the CD4 CTLs we observed in colitis are the same population/subset as recently described among CD4 IEL (51), both cells highly express CRTAM and possess killing function utilizing cytotoxic granules. Furthermore, CD4 CTL as Eomes-expressing CD4^+^ T cells are involved in late-onset EAE and may also have a major role in the progressive state of multiple sclerosis in humans (47). Eomes-deficient CD4^+^ T cells failed to induce late-onset EAE, suggesting that CD4 CTLs are responsible for this pathogenesis and could be target cells for therapeutic intervention.

## Conclusion

CD4 CTLs positively and negatively function in various types of peripheral inflammation sites, and affect both protective and pathogenic immunity. These disparate outcomes could be due to the Ag specificity of the CD4 CTLs. In the case of protection from viral infection, CD4 CTLs may be specific for viral Ag(s), whereas in the case of inducing autoimmunity, such as in colitis or EAE, these cells could be specific for microbiota in the intestine or self Ag(s), such as myelin basic protein. It will be important to investigate and understand the mechanisms of differentiation and function of CD4 CTL, particularly for promoting antiviral and antitumor immunity for host protection, as well as for effective intervention and therapy for autoimmune diseases.

## Author Contributions

AT performed experiments, and AT and TS wrote the manuscript.

## Conflict of Interest Statement

The authors declare that the research was conducted in the absence of any commercial or financial relationships that could be construed as a potential conflict of interest.
